# Prospective assessment of the gastroesophageal microbiome in VLBW neonates

**DOI:** 10.1186/1471-2431-13-49

**Published:** 2013-04-05

**Authors:** Vladana Milisavljevic, Meena Garg, Ivan Vuletic, Jeff F Miller, Lauren Kim, Tina D Cunningham, Imke Schröder

**Affiliations:** 1 Department of Pediatrics, Cedars-Sinai Medical Center, 8700 Beverly Boulevard, NT, Suite 4311, Los Angeles, CA, 90048, USA; 2 Department of Pediatrics, Neonatal Research Center, David Geffen School of Medicine, University of California Los Angeles, Los Angeles, CA, USA; 3 Department of Microbiology, Immunology and Molecular Genetics, David Geffen School of Medicine, University of California Los Angeles, Los Angeles, CA, USA

**Keywords:** Colonization, GI microbiome, Neonates, 16S rDNA

## Abstract

**Background:**

The distal GI microbiota of hospitalized preterm neonates has been established to be unique from that of healthy full-term infants; the proximal GI, more specifically gastroesophageal colonization has not been systematically addressed. We prospectively evaluated early colonization of gastroesophageal portion of the GI tract of VLBW infants.

**Methods:**

This study involved 12 infants admitted to a level III NICU with gestational age (GA) 27 +/− 0.5 weeks and birth weight 1105 +/− 77 grams**.** The gastroesophageal microbial flora was evaluated using 16S rDNA analysis of aspirates collected in a sterile manner during the first 28 days of life.

**Results:**

Bacteria were detected in 9 of the 12 neonates. *Ureaplasma* was the dominant species in the first week of life, however, staphylococci were the predominant bacteria overall. By the fourth week, Gram (−) bacteria increased in abundance to account for 50% of the total organisms. Firmicutes were present in the majority of the neonates and persisted throughout the 4 weeks comprising nearly half of the sequenced clones. Noticeably, only two distinct species of *Staphylococcus epidermidis* were found, suggesting acquisition from the environment.

**Conclusions:**

In our neonates, the esophagus and stomach environment changed from being relatively sterile at birth to becoming colonized by a phylogenetically diverse microbiota of low individual complexity. By the fourth week, we found predominance of Firmicutes and Proteobacteria. Bacteria from both phyla (CONS and Gram (−) organisms) are strongly implicated as causes of hospital-acquired infections (HAI). Evaluation of the measures preventing colonization with potentially pathogenic and pathogenic microorganisms from the hospital environment may be warranted and may suggest novel approaches to improving quality in neonatal care.

## Background

While transitioning from a relatively sterile intrauterine environment, neonates become colonized with a complex microbial population. Common environmental exposures influencing colonization include maternal vaginal, fecal, or skin microbiota, as well as breast milk or formula feedings, and result in the development of the intestinal microbiome. In contrast to healthy babies, preterm infants have prolonged hospital stays in the neonatal intensive care unit (NICU), and therefore are exposed to microorganisms specific to the hospital environment. Very low birth weight (VLBW) infants frequently experience hospital-acquired infection (HAI) during prolonged hospitalization, which significantly contributes to their mortality and morbidity. The GI microbiota derived from stool samples of hospitalized, preterm neonates was shown to be different from that of healthy, full-term neonates [1,2]. Examinations of the preterm neonatal fecal microbiota demonstrated a low biodiversity and strong prevalence of *Staphylococcus* and *Pseudomonas* species with increasing age of the infant [3-5]. Preterm neonates have an immature immune system, mucous membranes and GI tract; immature intestinal epithelial cells may display exaggerated inflammatory responses to both commensal and pathogenic bacteria [6,7]. In neonates bacteria may be more likely to translocate across the GI epithelium to organs and tissues, thereby increasing the risk for systemic infections [8]. The exact mechanisms of such bacterial translocation are still not well understood.

The proximal GI tract can be viewed as a likely “point of entry”, however, to date the microbiota of the proximal GI tract in neonates has not been systematically assessed. In adults, colonization of the proximal GI tract by pathogens has been associated with increased incidence of postoperative sepsis [9]. We hypothesized that in the NICU environment opportunistic and pathogenic bacteria, including *S. epidermidis*, influence the formation of the early microbiome of the gastroesophageal portion of proximal GI tract of preterm neonates. The objective of this study was to prospectively investigate the early acquisition of the gastroesophageal microbiome in VLBW neonates using 16S rDNA analysis.

## Methods

### Patients

All subjects enrolled in this study were inborn preterm neonates < 32 weeks gestational age with a birth weight <1500 g. They were born and admitted to either the Mattel Children’s Hospital UCLA or Santa Monica UCLA Medical Center NICU over a period of six consecutive months (11-1-09 to 4-30-10). Design of both NICUs is a multi-patient, open bay ward. Exclusion criteria were GA >32 weeks, birth weight >1500 g, lethal congenital malformations or outborn neonates. Neonates with non-lethal congenital anomalies were not excluded. All neonates were enrolled in the study on day 1 of life. Serial proximal GI aspirates were collected for the first 28 days of life and analyzed by week of life. Nasogastric (NG) tube was placed by the bedside nurse under clean conditions after hand washing and appropriate measurements. During the study period, use of gloves was in the guidelines, however, not necessarily always exercised (this has changed since, to the mandatory use of gloves). Correct placement of the NG tube was established by auscultation after injecting 3–5 cc of air. Gastric content has been checked every three hours; if the infant was intermittently feeding, prior to the feeding, if the patient on continuous feedings, every 3 hours during feeding. In some cases the pH of gastric content was tested to indicate correct placement. For this study purpose, the bedside nurses were instructed to save any gastric aspirate obtained when they checked for residuals. The aspirates were placed on ice and stored in the NICU specimen refrigerator. A member of study team was paged to pick up the samples as soon as collected for further analysis. NG tubes were changed daily. Precautions were exercised that, once collected, samples are handled under sterile conditions. Demographic and clinical data were obtained from review of medical records.

### Ethics statement

The UCLA Institutional Review Board approved the study. Written informed consent was obtained from the parents of all patients shortly after delivery.

### Molecular analysis

The composition of the gastroesophageal microbial flora was surveyed using 16S rDNA analysis. Briefly, 1 μl of the collected aspirates was directly added to PCR reactions. Control reactions were spiked with *E. coli* DNA to control for possible acidification of the reaction mix. Universal bacterial 16S rDNA primers 8F (5^′^-AGAGTTTGATYMTGGCTCAG) and 1510R (5^′^-TACGGYTACCTTGTTACGACTT) were used for 16S rDNA amplification. The high fidelity, rapid enzyme Phire polymerase (NEB) was used for all amplifications. Amplification reactions were done as follows: 3 min at 98°C, 25 cycles of 30 sec at 98°C, 20 sec at 55°C, and 1 min at 72°C, concluded with 5 min at 72°C. PCR reactions were limited to 25 cycles to minimize artifacts potentially caused by Phire polymerase. PCR products were agarose gel purified, TOPO cloned into plasmid pCR2.1, and transformed into *E. coli* TOP10 (Invitrogen). Plasmid inserts were sequenced by Beckman Coulter Genomics. Initially 8 clones were submitted for sequence analysis per positive sample. If the results contained consistently the same type of bacterial species, we assumed that we sequenced the sample to completion. However, if the sequencing results contained several distinct bacterial species, more clones were sent out for sequencing until no new species sequence was obtained (Table 1). If the sequence was >99% identical to a bacterial species in the database, we assigned it the name given in the database. All sequences were submitted to the National Center for Biotechnology Information (NCBI) database.

**Table 1:** Bacterial species and sequenced clones by week per infant with positive aspirates

**Infant**	**Week 1**	**Week 2**	**Week 3**	**Week 2**
1	0	4 (12)	2 (8)	0
2	5 (24)	0	0	0
3	4 (12)	0	0	0
4	0	0	2 (8)	0
5	2 (8)	0	2 (8)	3 (12)
6	0	3 (12)	0	0
7	0	2	2 (8)	0
8	0	2	4 (12)	3 (12)
9	4 (12)	0	3 (12)	6 (30)

The first number indicates the distinct bacterial species that were identified, the number in brackets clones that had been sequenced.

### Bacterial isolates

Collected aspirates were directly plated on tryptic soy agar (Difco) and bacterial colonies were purified by re-streaking on the same medium after overnight incubation at 37°C under aerobic conditions. Bacterial isolates were identified by 16S rDNA sequence analysis and their sequence was submitted to the National Center for Biotechnology Information (NCBI) database.

### Data analysis

The DNA sequences of each clone set were compared to each other to eliminate redundant sequences. Unique sequences were blasted against the non-redundant nucleotide database. Most sequences matched 16S rRNA sequences in the database with E values of 0 and were designated to be the same sequence. For the construction of the phylogenetic tree, sequences were retrieved from the NCBI database. Two 16S rRNA sequences with poor matches to the database were submitted to NCBI. Sequences were aligned using ClustalW, and the phylogenetic tree was constructed with the TREECON software using the Kimura 2 algorithm.

Descriptive statistics were computed for the prenatal and postnatal characteristics from the 12 subjects. The primary outcome of interest was whether or not gastroesophageal aspirates tested positive for bacteria. Since our sample size was very small, Fisher’s exact test was used to evaluate the association between clinical variables potentially affecting colonization and the presence or absence of bacteria during the first four weeks of life. Clinical variables evaluated were delivery mode (vaginal or cesarean section), rupture of membranes, chorioamnionitis, type of feedings (formula, breast milk, breast milk plus formula), use of antibiotics, use of famotidine and use of respiratory support. We performed analysis using SAS/STAT software (version 9.2, SAS Institute Inc, Cary, NC).

## Results

### Clinical characteristics of the neonatal cohort

Clinical characteristics, including demographic and clinical data of 12 study subjects are summarized in Tables 2 and 3. Data for 2 of 12 infants were not available after week two of study due to transfer to another facility in one, and a death unrelated to sepsis in the other case.

**Table 2:** Clinical characteristics for the 12 subjects enrolled in the study

**Summary of demographic and clinical data**	
**Prenatal characteristics**	**n = 12 infants**
Gestational age in weeks	29.2 ± 0.7* (24–32)
Birth weight in grams	1140 ± 72* (580–1390)
Male sex	8 (67%)
Cesarean section	10 (83%)
Maternal antenatal steroids	12 (100%)
Maternal antenatal antibiotics	6 (50%)
Maternal chorioamnionitis	2 (17%)
Prolonged rupture of membranes	4 (33%)
**Postnatal characteristics**	
RDS requiring intubation/surfactant	9 (75%)
RDS requiring CPAP - high flow nasal cannula	3 (25%)
Umbilical artery or vein catheterization	11 (92%)
PICC line placement	12 (100%)
Antacid use (famotidine)	6 (50%)
Antibiotics in first 48 hrs	11 (92%)
Positive blood culture (late onset sepsis)	1
Positive tracheal culture	3 (25%)

*Mean ± SEM.

**Table 3:** Enteral nutrition and clinical variables by week of life

	**Week 1 (n = 12)**	**Week 2 (n = 12)**	**Week 3 (n = 10)**	**Week 4 (n = 10)**
**Enteral nutrition**				
Enteral feeds	7	9	9	8
Breast milk feeds only	2	7	9	7
Formula feeds only	0	0	0	1
Breast milk + formula feeds	5	2	0	0
Full enteral feedings	1	3	4	4
**Clinical variables**				
Famotidine	4	5	4	6
Antibiotics	12	2	2	5
NG Tube/Repogle	12	12	12	12
ETT	9	4	5	5
CPAP/HFNC	3	6	3	1
Nasal cannula O_2_	0	2	2	4

### Statistical analysis

Analysis of all clinical data using Fisher’s exact test showed no association between any of the evaluated clinical variables (delivery mode, rupture of membranes, chorioamnionitis, type of feedings, uses of antibiotics, famotidine and respiratory support) and the presence or bacteria distributed in the four phyla identified in our samples (p-values >0.05).

### Gastroesophageal microbiome evaluated at phyla level

**Figure 1:**
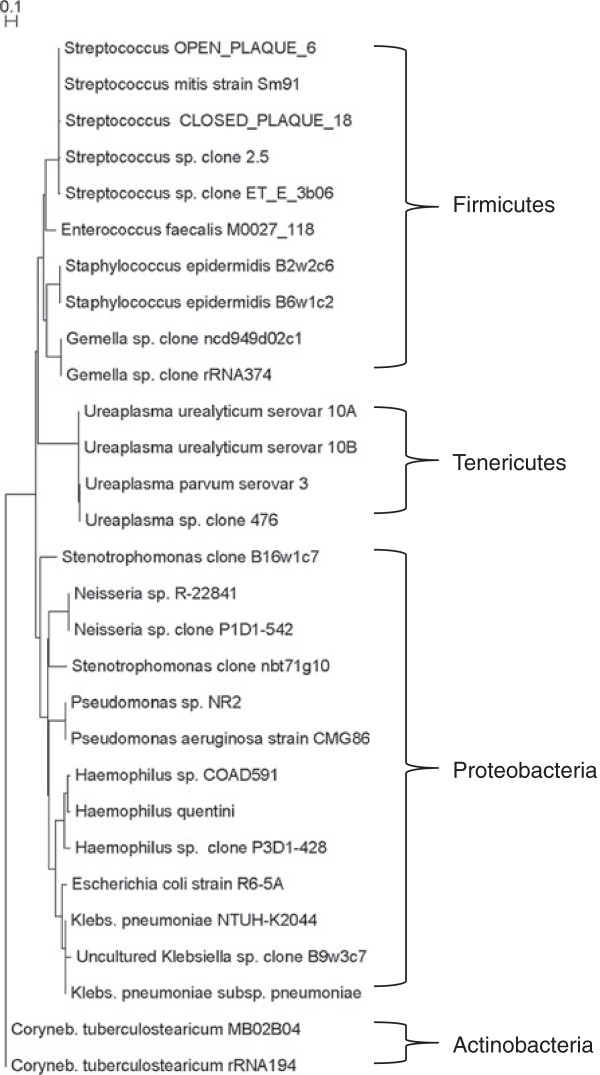
Phylogenetic relationships of bacteria isolated from the gastroesophageal aspirates.

Over four weeks of our study a total of 108 gastroesophageal aspirates were collected from the patient cohort. The range of gastroeasohageal aspirates obtained was 4–26 per infant, however, not all the samples obtained from the infant with 26 samples were analyzed, as they were days when the patient had several samples. When there were duplicate samples obtained the same day from an infant, only one was analyzed. Ninety-five samples were analyzed by PCR using universal primers to the 16S rDNA gene. Twenty-five cohort samples tested positive for bacteria and the PCR products were cloned. A total of 190 clones were sequenced identifying 4 phyla containing 13 bacterial species with 29 subspecies (Figure 1). In 3 of the 12 infants no bacteria were detectable through the 4 weeks of the study (Table 4).

**Table 4:** Summary of all the bacterial species identified based on 16S rDNA sequence analysis of upper GI aspirates in the individual VLBW study subjects

		**Infant number**											
**Genus**	**Species**	**1**	**2**	**3**	**4**	**5**	**6**	**7**	**8**	**9**	**10**	**11**	**12**
*Staphylococcus*	*S. epidermidis* B2w2c6**		x		x			x	x	x			x
	*S. epidermidis* B6w1c2**				x			x	x	x			x
*Streptococcus*	*S. mitis* Sm91										x		
	*S.* clone ET_E_3b06												x
	*S.* clone 2.5												x
	*S.* Closed Plaque 18												x
	*S.* Open Plaque 6										x		
*Gemella*	*G.* clone rRNA374												x
	*G.* clone ncd949d02c1												x
*Enterococcus*	*E. faecalis* B6w1c4**				x								x
*Ureaplasma*	*U. parvum* clone 476		x		x	x							
	*U. parvum* serovar 3				x	x							
	*U. urealyticum* ser. 10 A												x
	*U. urealyticum* ser. 10 B												x
*Haemophilus*	*H. quentini*										x		
	*H.* COAD591										x		
	*H.* P3D1-428										x		
*Stenotrophomonas*	S. clone nbt71g10					x			x				
	*S.* B16w1c7*												x
*Klebsiella*	*K. pneumoniae* MGH						x						
	*K. pneumoniae* NTUH-K2044						x						
	*K. pneumoniae* B9w3c7*										x		
*Pseudomonas*	*P. aeruginosa* CMG860		x										
	*P*. NR2		x										
Escherichia	*E. coli* R6-5A							x					
*Neisseria*	*N.* clone P1D1-542												x
	*N.* R-22841												x
*Corynebacterium*	*C. tuberculostearicum* rRNA194		x		x								
	*C. tuberculostearicum* MB02B04		x										

*Species were identified in this study; **bacteria were isolated in pure culture.

**Figure 2:**
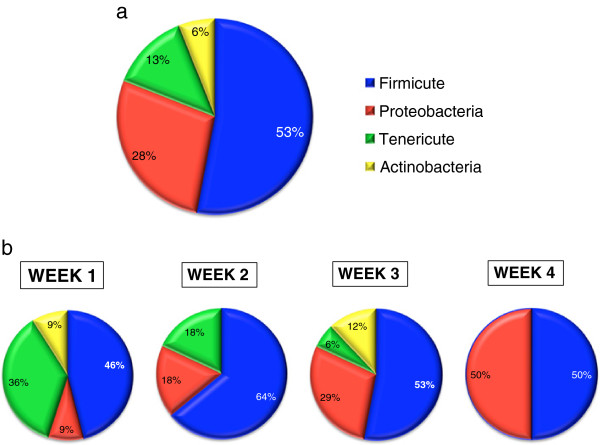
**The phylum abundance of bacteria identified in the gastroesophageal aspirates of VLBW neonates. a.** Over 4 weeks of study. **b.** Per week after birth.

In 9 infants with detected bacteria, Firmicutes constituted the predominant phylum followed by Proteobacteria, Tenericutes and Actinobacteria (Figure 2a). Firmicutes persisted during the course of the 4-week study, comprising approximately 50% of all clones. Tenericutes, which made up a relatively large population in our samples during the first week of life, markedly diminished and disappeared by week 3. In contrast, Proteobacteria, constituting a relatively minor population in the first week of life, gradually increased in number over 4 weeks of the study to account for ~ 50% of all clones (Figure 2b).

### Gastroesophageal microbiome evaluated at genus level

**Figure 3:**
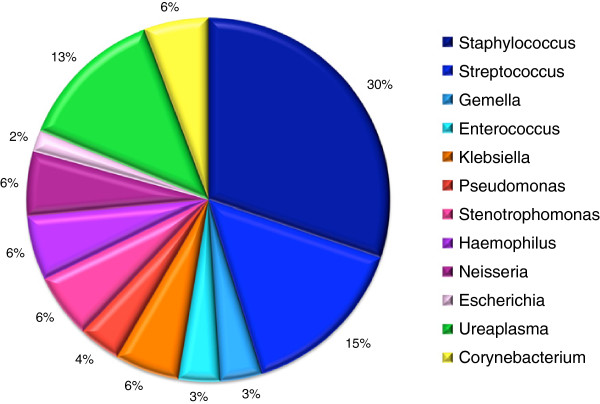
The genera diversity of bacteria identified in the gastroesophageal aspirates of VLBW neonates.

A summary of all the bacterial species identified from the gastroesophageal aspirates in 9 of 12 VLBW study subjects is shown in Table 4. Staphylococci were the most commonly detected bacterial species comprising about one third of all sequenced clones (Figure 3). We detected the presence of two reoccurring species of staphylococci, i.e., *Staphylococcus epidermidis* B2w2c6 (in 6/9 infants positive for bacteria) and *S. epidermidis* B6w1c2 (in 5/9 infants) (Table 4). We isolated both *Staphylococcus* species by plating gastric samples directly onto tryptic soy agar and submitted their 16S rDNA sequence to the NCBI database.

*Streptococcus* and *Ureaplasma* species were the second most abundant bacteria within the sequenced bacterial species library (Figure 2). In contrast to *Staphylococcus*, the *Streptococcus* genus consisted of 5 species, all of which were detected in two infants (Table 4 and Figure 1). The *Streptococcus* species included the alpha-hemolytic *S. mitis* Sm91, and streptococci previously identified as part of the oral bacterial microbiome ([10], Parahitiyawa NB, *et al*, unpublished, submitted 2009 to the INSDC). *Gemella,* a close relative of *Staphylococcus* and normally found on human mucous membranes, was detected in week 4 only in one infant.

*Ureaplasma* species were predominantly present during the first week of life (Table 4, Figure 3)*.* Two *Ureaplasma parvum* and two *Ureaplasma urealyticum* serovars were identified in 4 neonates. It is likely that all *Ureaplasma* bacteria were transmitted during delivery or acquired *in utero* via infected amniotic fluid [11]. Their marked absence in the later weeks of life suggests that *Ureaplasma* fails to persist in the gastric environment and/or was eliminated by antibiotic treatment.

**Figure 4:**
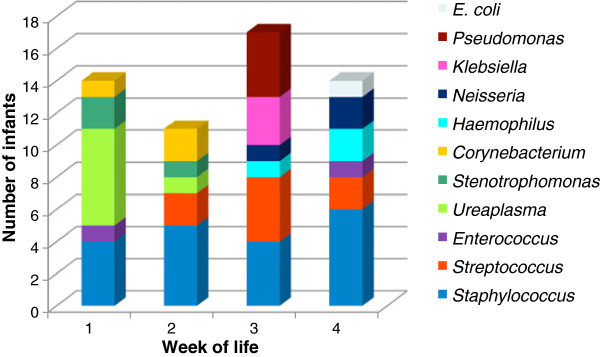
Number of infants colonized by distinct bacteria during the 4 weeks of study.

Other, less abundant, bacteria detected in the first two weeks of life include *Stenotrophomonas*, *Corynebacterium tuberculostearicum* and *Enterococcus faecalis* species (Figure 4). *E. faecalis* B6w1c4 was isolated from a gastric aspirate sample by plating on tryptic soy agar.

The overall percentage of Gram (−) organisms including *Neisseria, Haemophilus, K. pneumoniae, P. aeruginosa* and *E. coli* species increased from 9% in the week 1 to 50% of all the organisms in the week 4 (Figures 2b and 4).

## Discussion

This report illustrates the phylogenetic diversity of the gastroesophageal microbial flora in VLBW infants from birth to 4 weeks of age using 16S rDNA analysis of obtained aspirates. All the neonates were enrolled during a period of 6 month. The gastroesophageal portion of the GI tract was chosen for the study since naso/oro-gastric tubes are placed in all VLBW infants immediately after birth, and may be one of the initial entryways of colonization; stool output is often delayed for up to 5–7 days after birth. We demonstrate that in the first 4 weeks of life preterm infants acquire a relatively low complexity bacterial community ranging from 2 to 13 distinct bacterial species (Table 4, Figure 3). This portion of GI tract is not always sterile immediately after birth, as we detected *Ureaplasma* ssp. and *S. epidermidis* in the early samples. Maternal Ureaplasma colonization has been associated with preterm labor and chronic lung disease in neonates [12]. In a study by Oue *et al.*[13], the authors found that detection of *Ureaplasma* species in gastric fluids was associated with subsequent respiratory colonization, likely promoting the onset of bronchopulmonary dysplasia.

Staphylococci were the predominant organisms during the first four weeks of life in the gastroesophageal microbial flora of infants in our study. This suggests an early and constant exposure of affected infants and/or the capability of Firmicutes to persist despite the use of antibiotics. While 16S rRNA genes sequencing identified two distinct *S. epidermidis* species only, species resolution may require the analysis of additional core genes. However, it is intriguing to consider that populations of the those two *S. epidermidis* were identified in 50% of the cohort over the 6 months, implying acquisition from the hospital environment rather than from the mother’s skin. *S. epidermidis* is a normal inhabitant of skin and mucous membranes, however, it is a potential pathogen in patients that are immunocompromised or have indwelling foreign bodies. Since the late 1970s, studies have demonstrated that coagulase-negative staphylococci (CONS) are the most common cause of late onset sepsis among NICU low birth weight neonates resulting in 50-70% of all bloodstream infections [14-16]. In the study by Stoll *et al.*, CONS caused late onset sepsis in 48% of VLBW infants [17]. Hemolytic peptide δ-toxin, the only identified toxin produced by *S. epidermidis*, has been associated with necrotizing enterocolitis (NEC), a major morbidity factor in preterm neonates [18]. The pathogenesis of foreign-body-associated infections caused by *S. epidermidis* is characterized by its ability to colonize polymer surfaces and form biofilms. Bacteria from the patient’s skin and mucous membranes, or acquired from hands of the hospital staff, can contaminate the polymer during placement of the foreign body or its subsequent care. So far, most studies have focused on intravascular catheters. Hurrell *et al.* observed biofilm formation inside naso/oro-gastric tubes, and Enterobacteriaceae comprised the major organisms cultured from biofilms [19,20]. Mehall *et al.* isolated *S. epidermidis* and methicillin-resistant *S. aureus* amongst other bacteria from naso/oro-gastric tubes [21,22]. Naso/orogastric tubes are used to “vent” the stomach, assess gastric residuals and feeding readiness, and for gavage feedings until infant is able to adequately breast/bottle feed. In the case of extremely premature neonates, this can last 10 weeks or longer. Healthcare providers, whose hands are colonized by CONS, still frequently place naso/oro-gastric tubes without using sterile techniques, since there are currently no clear handling guidelines recommended.

Proteobacteria constituted a relatively minor population in the first week of life, but gradually increased in number over the 4 weeks of the study to comprise ~50% of all clones (Figures 2b and 4). The increasing abundance of Gram (−) negative bacteria such as *Haemophilus*, *Stenotrophomonas*, *Klebsiella*, *Pseudomonas*, *Escherichia*, and *Neisseria* is particularly concerning since these organisms are frequent pathogens causing HAI [23]. It is likely that these bacteria were acquired from the hospital environment, since they are not known to be present in formula or breast milk feedings. However, we cannot exclude possibility of the initial undetected low-level colonization that increased with time. Infections caused by these organisms are closely associated with their ability to produce biofilms.

We did not find statistically significant relationships between the presence of distinct bacteria and the clinical data collected for each infant. However, our study evaluates a small number of neonates, and the influence of certain clinical practices on bacterial colonization cannot be excluded. In healthy term newborns, the gut microbiota and its evolution over time vary from individual to individual [24]. A twin study that monitored bacterial composition suggests that the initial stages of the infant’s GI flora are largely dependent on the specific bacteria to which a baby is exposed [24]. Schwiertz *et al.* found that preterm infants acquired a low complexity bacterial community after birth, and as a result of hospitalization, developed a similar strain composition over time [1].

Evidence from this and other reports suggests that a shared environment (such as multi patient, open bay ward NICU) can be a major factor. Further studies with an increased cohort size are warranted to evaluate a possible link between the developing GI microbiome and neonatal morbidities. A larger study may support new diagnostic and management approaches in the NICU, as well as suggest interventions for prevention of colonization with potentially pathogenic and pathogenic organisms.

## Conclusions

The gastroesophageal portion of GI tract of VLBW neonates is not always sterile at birth. In the first four weeks of life, VLBW infants managed in the NICU developed a phylogenetically diverse gastroesophageal microbiota of low complexity. In this study, *S. epidermidis* is overall the predominant organism colonizing gastroesophageal portion of the GI tract throughout the first four weeks of life. By the fourth week of life, the gastroesophageal microbiome of the VLBW infants changed to predominantly compose of Firmicutes and Proteobacteria. Bacteria from both phyla, CONS and Gram (−) organisms, can be biofilm producers and are strongly implicated as causes of hospital-acquired infections (HAI). Our study supports the notion that microorganisms present in the hospital environment colonize gastroesophageal portion of the GI tract of preterm neonates, and evaluation of measures that would decrease colonization of immunocompromised patients, such as preterm neonates, with potentially pathogenic and pathogenic bacteria is warranted.

## Abbreviations

16S rDNA: 16S ribosomal DNA; VLBW: Very low birth weight; NICU: Neonatal intensive care unit; PPROM: Preterm premature rupture of membranes; PROM: Premature rupture of membranes; DOL: Day of life; GI: Gastrointestinal, RDS, respiratory distress syndrome, LOS, late onset sepsis; CONS: Coagulase-negative staphylococci

## Competing interests

The authors declare that they have no competing interests.

## Authors’ contributions

VM, MG, IM: Substantial contributions to conception and design, acquisition of data, or analysis and interpretation of dat. IV, LK, TDC: Acquisition of data, or analysis and interpretation of data. VM, IM, MG, JFM: Drafting the article or revising it critically for important intellectual content. All authors read and approved the final manuscript.

## Pre-publication history

The pre-publication history for this paper can be accessed here:

http://www.biomedcentral.com/1471-2431/13/49/prepub
